# The May Meetings at Atlanta

**Published:** 1896-07

**Authors:** 


A Monthly Review of Medicine and Surgery.

EDITORS:

THOMAS LOTHROP, M. D. - - WM. WARREN POTTER, M. D.

All communications, whether of a literary or business nature, should be addressed to the managing editor: 284 FRANKLIN STREET, BUFFALO, N. Y.

VOL. XXXV. JULY, 1896. No. 12.

### THE MAY MEETINGS AT ATLANTA.

THERE is no more delightful portion of our country in May than the midsouth. In that season of the year there is a freshness of vegetation and a balminess of atmosphere saturated with the perfume of spring blossoms, all conspiring to make a journey to the region referred to specially attractive at that time. Hence it is not surprising that the group of medical meetings held at Atlanta last May was not only well attended but that it proved above the average in scientific excellence.

### AMERICAN ACADEMY OF MEDICINE.

The series began with the meeting of this society, held May 2d and 4th, and we may truthfully affirm that this was one of the best meetings ever held by this distinguished body of physicians. The work doing by the academy in advancing the cause of higher medical education is not easy to estimate, but it is to be commended in the highest degree. Nearly all the papers and discussions at the late meeting were shaped in that direction and will exert a beneficial influence.

The erudite and indefatigable secretary of the academy, Dr. Charles McIntire, of Easton, Pa., deserves special praise for the methodical and skilful manner in which he is conducting the affairs of the academy. The *Bulletin* of the academy, which he so ably conducts, is becoming a magazine of influence, and every physician interested in the advancement of medical education in the United States, whether a member of the academy or not, should become a subscriber. In its columns will be found material nowhere else published, and as a work of reference it is invaluable, showing the progress making in the medical reform to which it is specially devoted.

### NATIONAL CONFEDERATION STATE MEDICAL EXAMINING AND LICENSING BOARDS.

The next body to convene was the Confederation of State Medical Examining and Licensing Boards, which met on Monday, May 4th. Though this organisation has had a nominal existence for four or five years, it is only now beginning to make itself felt according to its importance. Through a cohesive union of the several state boards in a national confederation much good may result in the direction of facilitating work, improving results and unifying and standardising methods.

The Atlanta meeting of this confederation, the proceedings of which we publish in another column, did much toward laying the foundation for a usefulness such as is outlined in the foregoing paragraph. One of the things which it certainly bids fair to accomplish is the preventing of inconsiderate acting in regard to a national examining board. Such a board certainly is not necessary under present conditions, and it clearly could have no right to endorse the lieenses of state boards, thereby making them valid throughout the United States, until the states themselves establish a minimum level of requirements.

### THE ASSOCIATION OF AMERICAN MEDICAL COLLEGES.

The seventh annual meeting of this association was also held at Atlanta, May 4, 1896. Its first session was held in the morning in conjunction with the American Academy of Medicine and the Confederation of State Boards of Medical Examiners for the purpose of discussing methods of medical education in a series of papers prepared under the direction of the American Academy of Medicine. These papers will appear in full in the August issue of the *Bulletin* of the academy and should be read by every person interested in the subject.

At the afternoon session the president, Dr. William Osler, delivered his address, the report of the committee on syllabus was presented, the financial report of the secretary was read and other important business transacted. The election of officers resulted in bringing to the presidency Dr. J. M. Bodine, of Louisville, who is the dean of the University of Louisville, and a most capable and interested officer. The interests of the college association will be well served at the hands of Dr. Bodine.

### THE AMERICAN MEDICAL ASSOCIATION.

This body convened on Tuesday, May 5th, and was presided over by Dr. Beverly Cole, of California. The addresses of welcome by Dr. John M. Ridley, of LaGrange, and John Temple Graves, Esq., of Rome, were two of the cleverest specimens of welcoming oratory to which we ever listened, and we only regret that limited space prevents their reproduction in these columns. Dr. Cole stirred the depths somewhat with his address, but it was reserved for Dr. Senn to furnish the *ad captandem* when he flayed the specialists, particularly the laryngologists and the gynecologists, by his "talk to the galleries" in his address on surgery. 'Tis ever thus with the general surgeon when he gets an opportunity to berate the specialist in pelvic and abdominal surgery before an audience from which there is no talking back.

The attendance, as shown by the registration, was fairly good in numbers and excellent in quality. The arrangements for the accommodation of guests were never better, while the hospitalities were royal, all under the direction of the chairman of the committee of arrangements, Dr. Willis F. Westmoreland.

### THE SOUTHERN RAILWAY EXCURSION.

A fitting denouément to the hard work of the week was the excursion tendered by the officers of the Southern Railway to a number of invited guests, under the direction of Dr. C. M. Drake, chief surgeon. A train consisting of two directors' cars, three Pullman sleepers and a dining coach left Atlanta, Friday evening, May 8th, and landed the tourists at Lookout Mountain Saturday morning in season for breakfast. Leaving Lookout at 9.30 A. M., a call was made at the Tate Epsom spring via Knoxville, where a visit was paid at 5.30 P. M., and Hot Springs, N. C., was reached at 8 P. M., when dinner was served and where a delightful night was spent. Next morning a bath in the natural hot water was enjoyed by most of the guests, after which breakfast was served and the train moved forward to Asheville, which it reached in season for luncheon on Sunday. Here, too, post-prandium speeches were the order, when a number of the guests expressed their opinion of the treatment they were receiving at the hands of the Southern Railway. Messrs. W. A. Vaughan, H. M. Aiken and F. K. Huger, division superintendents, accompanied the party over their respective divisions and received the thanks of the tourists for their attentions. Again boarding the train at 3 o'clock onward it sped over the mountains towards Salisbury, where at 8 o'clock the excursionists divided, some going northward and the remainder returning to Atlanta, reaching there Monday morning for breakfast. Before separating, however, the tourists presented Dr. Drake with an address and a suitable memento, to be arranged by Drs. A. Walter Suiter and Frank Woodbury as a committee. Thus ended one of the most delightful medical gatherings that it has been our privilege to enjoy.

